# Role of supplemental foods and habitat structural complexity in persistence and coexistence of generalist predatory mites

**DOI:** 10.1038/srep14997

**Published:** 2015-10-09

**Authors:** Alberto Pozzebon, Gregory M. Loeb, Carlo Duso

**Affiliations:** 1 Department of Agronomy, Food, Natural Resources, Animals and Environment, University of Padova, Italy; 2 Department of Entomology, New York State Agricultural Experiment Station, Cornell University, Geneva, NY, USA

## Abstract

Plant traits can influence the interactions between herbivore arthropods and their natural enemies. In these interactions generalist predators are often present, preying on herbivores and also on other arthropods in the same trophic guild. Variation in the strength of intraguild predation (IGP) may be related to habitat structural complexity and to additional resources outside the narrow predator-prey relationship. In this paper we study the food web interactions on grape, which involves two generalist predatory mites. We evaluated the effects of grape powdery mildew (GPM) as supplemental food, and habitat structural complexity provided by domatia. The inoculation of GPM resulted in higher predatory mite densities and reduced the negative impact of unfavorable leaf structure for one species. Access to domatia was the main factor in promoting population abundance and persistence of predatory mites. Access to domatia and GPM availability favored the coexistence of predatory mites at a low density of the intraguild prey. Our findings suggest that structural and nutritional diversity/complexity promote predatory mite abundance and can help to maintain the beneficial mites - plants association. The effect of these factors on coexistence between predators is influenced by the supplemental food quality and relative differences in body size of interacting species.

Omnivores, in particular generalist predators, can exploit resources at different trophic levels, and therefore may prey on other arthropods that are also natural enemies in the same trophic guild (i.e., intraguild predation, IGP)^1^,^2^,^3^. Intraguild predation can shape the outcome of biological control by multiple predator assemblages^4^,^5^,^6^,^7^. Theoretical studies predict that coexistence and thus persistence of intraguild predators are likely at intermediate levels of food resource availability and when the intraguild prey (IG-prey) is a superior competitor for the shared resource than the intraguild predator (IG-predator)^1^,^8^,^9^. Departures from these predictions have been found to be associated with the availability of trophic supplements outside the IGP modules and habitat structural complexity, among other factors. Intraguild predation can be influenced by supplemental resources outside the narrow predator-shared resource relationship^9^,^10^,^11^. Variation in the strength of intraguild predation may also be related to habitat structural complexity^5^,^12^,^13^. In particular, the presence of habitat structures, acting as refuges or reducing probability of encounter rate, can result in reduced predation pressure on IG prey. This would increase the possibilities for persistence of systems with intraguild predation^13^.

Intraguild predation is a widespread phenomenon among biological control agents of importance in several cropping systems^14^. Trophic supplements and habitat structural complexity have important effects on several generalist predators that can engage in IGP^13^,^15^. Supplemental food sources are important in maintaining the population of biological control agents in absence of their prey^16^,^17^. It is well documented that increased habitat structure created by tufts of non-glandular trichomes located at the conjunction of veins on the leaf blade (acarodomatia or domatia) mediate interactions among natural enemy arthropods, the plant and herbivores. During the last few decades various researchers have studied the role of leaf domatia in trophic interactions^18^,^19^. Domatia may benefit predators through different mechanisms including protection from higher order predation, capture of non-prey food such as pollen, and refuge from low humidity conditions^20^,^21^,^22^,^23^,^24^. Plants obtain benefits in terms of a reduced incidence of parasites^25^,^26^ with an increase in their fitness^27^,^28^. Domatia can play an important role in enhancing biological control of pests attacking economically important plants, such as grapevines^25^,^29^,^30^.

In this paper we were interested in quantifying the interacting effect of structural complexity and supplemental food resources on predator coexistence and persistence. We used two predatory mite species that are important biocontrol agents on grapevine, *Amblyseius andersoni* (Chant) and *Typhlodromus pyri* Scheuten. They can persist on grapevines with few or no mite prey potentially feeding on wind-borne pollen and plant-pathogenic fungi like Grape powdery mildew (GPM) *Erysiphe necator* Schw.^31^,^32^,^33^,^34^. Leaf traits of grapevines are often a better predictor of abundance of generalist predatory mites than the abundance of mite prey^35^,^36^. These predators are known to engage in reciprocal intraguild predation representing an interesting case-study within this framework^37^,^38^.

In this experiment we manipulated access to domatia and inoculation of grape powdery mildew (GPM) as a supplemental food source. We hypothesized that availability of a trophic supplement and structural complexity will promote predator persistence in the absence of prey. We also investigated if habitat structural complexity and a trophic supplement can affect IGP between predatory mites, promoting coexistence of predators. We hypothesized that these factors interact in a synergistic way thereby enhancing predatory mites persistence.

## Results

### Grape powdery mildew symptoms

**Figure 1:**
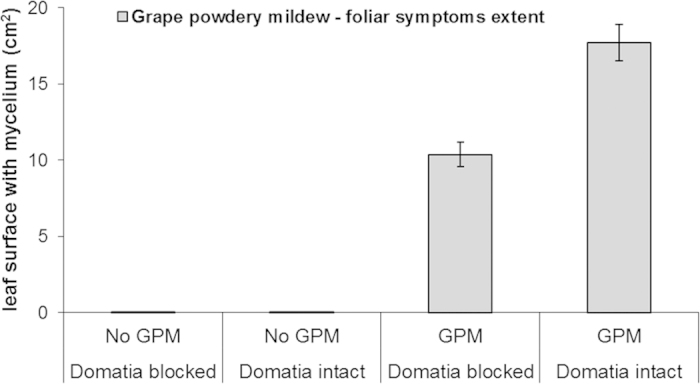
Levels of grape powdery mildew (mean ± standard error) as measured by surface area covered with mycelium on the bottom side of leaves of potted grape plants as a function of domatia manipulation and inoculation of GPM recorded at the end of the experiment.

Foliar symptoms of GPM on the underside of leaves reached higher levels on plants that received GPM inoculations compared to negligible GPM symptoms on the other plants in the experiment (Table 1, Fig. 1). For vines inoculated with GPM, those with open domatia had significantly more mycelium on leaves than vines with blocked domatia. The significant interaction between the domatia treatment and inoculation with GPM indicates the difference in GPM symptoms between open and blocked domatia (t_87.02 _= −5.13; *P* < 0.0001) while no differences were evidenced in un-inoculated plants (t_87.02 _= 0.01; *P*_ _= 0.99).

**Figure 2:**
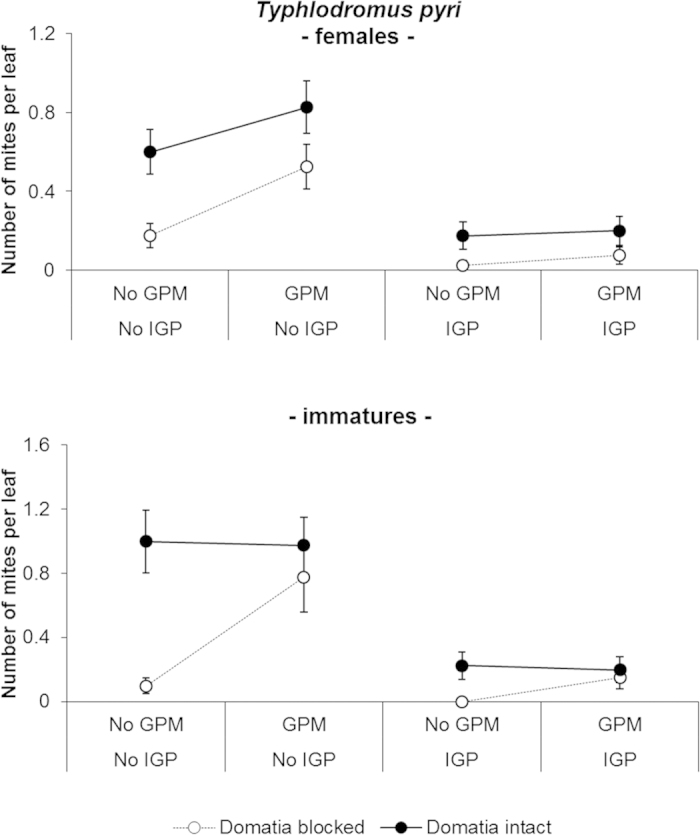
Effect of GPM inoculation, IGP and domatia availability on abundance per leaf of females and immatures of *Typhlodromus pyri* (mean ± standard error) observed at the end of the experiment.

*Typhlodromus pyri.* The densities of *T. pyri* increased from 1.6 mites/leaf (released density) to 2.15 mites/leaf (final density) during the experiment on treatments with open domatia and in absence of the competitor *A. andersoni* (Table 2, Fig. 2). Both immatures and females responded positively to GPM. An interaction between the IGP and domatia treatments was significant for motile forms and immatures (Table 2). Indeed, in absence of IGP the number of predatory mites was higher in the open domatia treatment relative to the blocked domatia treatment (motile forms: t_55 _= −6.61; *P* < 0.0001, immatures: t_52.35 _= −5.12; *P*_ _= 0.0002), whereas in the presence of *A. andersoni* the positive effects of domatia were not observed (motile forms: t_55 _= −2.21; *P*_ _= 0.181, immatures: t_52.35 _= −1.48; *P*_ _= 0.923; Fig. 2). However, proportionally the magnitude difference between open and blocked domatia was about the same with or without IGP (Fig. 2).

Another significant interaction occurred between domatia and GPM. Higher numbers of immatures and motile forms were observed in presence of GPM compared to leaves without GPM on leaves with blocked domatia (motile forms: t_55 _= −3.39; *P*_ _= 0.007, immatures: t_52.35 _= −3.36; *P*_ _= 0.008) while no differential effect for GPM was observed with open domatia (motile forms: t_55 _= 0.04; *P*_ _= 0.99, immatures: t_52.35 _= 0.33; *P*_ _= 0.99) (Table 2, Fig. 2).

**Figure 3:**
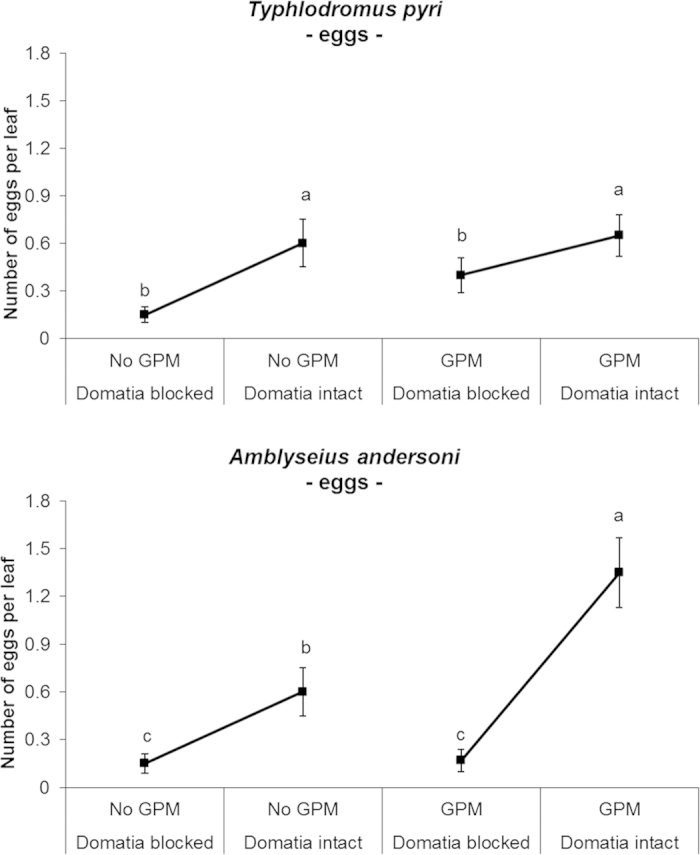
Effect of GPM inoculation and domatia availability on *Typhlodromus pyri* and *Amblyseius andersoni* eggs abundance per leaf (means ± standard error) observed at the end of the experiment. Only data from single species release treatments are showed in figure.

The number of eggs was positively affected by open domatia but not by the presence of GPM (Table 2, Fig. 3). The abundance of eggs was proportional to the number of females, with a similar eggs/female ratio among treatments (ranged from 0.76 to 1.00 eggs/female) (GPM: χ^2^ = 0.01, *P*_ _= 0.967; Domatia: χ^2^ = 0.39, *P*_ _= 0.531; Domatia*GPM: χ^2^ = 0.18, *P*_ _= 0.668).

**Figure 4:**
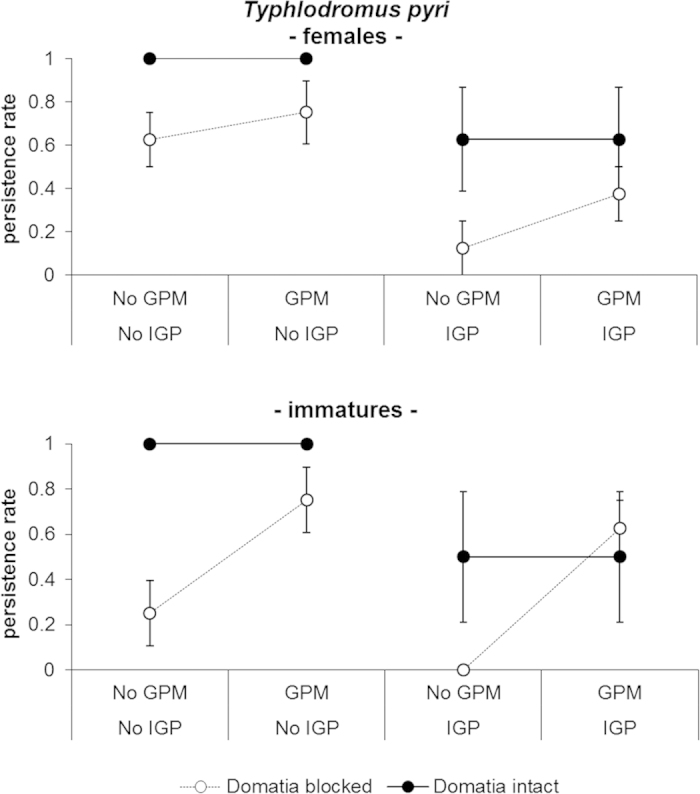
Effect of GPM inoculations, IGP and domatia availability on persistence of females and immatures of *Typhlodromus pyri* (mean ± standard error) observed at the end of the experiment.

A negative effect of IGP was observed on *T. pyri* persistence rate, while a positive effect was observed for domatia and GPM (Table 3, Fig. 4). A significant interaction between GPM and domatia was observed on *T. pyri* immatures and motile forms (Table 3). The effect of domatia was significant in absence of GPM (motile forms: t_23 _= 3.30; *P*_ _= 0.0189, immatures: t_23 _= 3.79; *P*_ _= 0.006), but not in presence of GPM (motile forms: t_23 _= 0.38; *P*_ _= 0.708, immatures: t_23 _= 0.47; *P*_ _= 0.642; Fig. 4). Vines with IGP, blocked domatia and without GPM represented the worst situation for *T. pyri* persistence and in this situation no immatures were found on leaves (Fig. 4).

### Amblyseius andersoni

**Figure 5:**
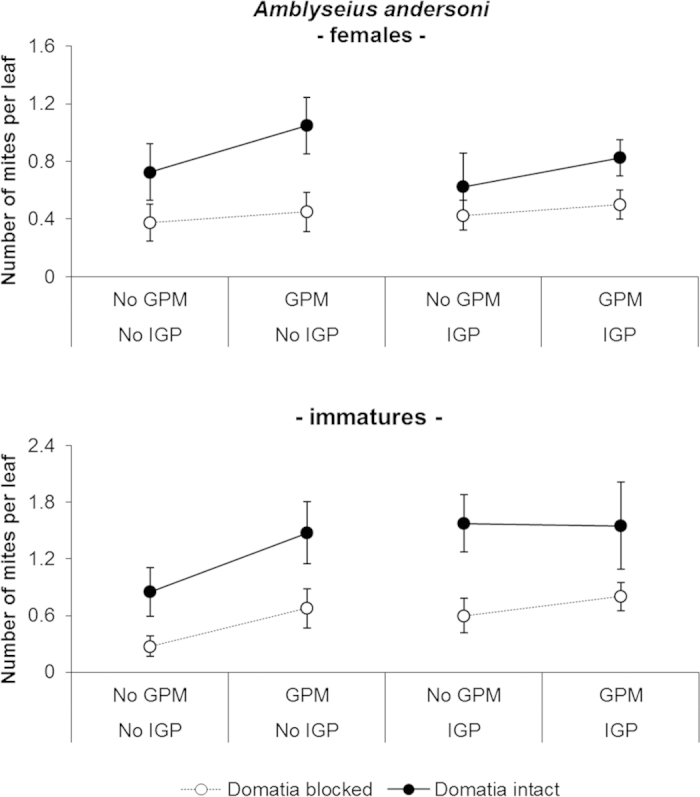
Effect of GPM inoculations, IGP and domatia availability on abundance per leaf of females and immatures of *Amblyseius andersoni* (mean ± standard error) observed at the end of the experiment.

*Amblyseius andersoni* successfully colonized its release treatments and at the end of the experiment the number of mites observed in treatments with open domatia was higher than initial densities (from 1.6 mites/leaf to 2.43 mites/leaf; Fig. 5). Domatia availability was the main factor promoting *A. andersoni* abundance, while less of an impact was observed for GPM infection, which was limited to motile forms (*P*_ _= 0.041) (Table 2). No effects of IGP were observed (Table 2; Fig. 5).

**Figure 6:**
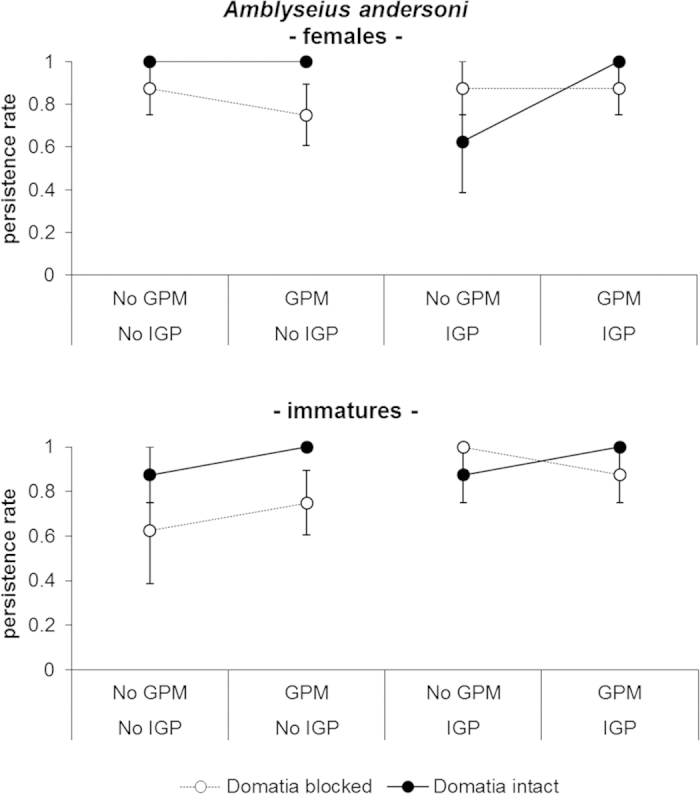
Effect of GPM inoculations, IGP and domatia availability on persistence of females and immatures of *Amblyseius andersoni* (mean ± standard error) observed at the end of the experiment.

GPM infection and access to domatia showed positive effects on egg abundance (Table 2, Fig. 3). The analysis of eggs densities indicated a significant interaction between the GPM and domatia treatments. When domatia were blocked the presence of GPM level did not result in a significant increase in eggs (t_28 _= −0.20; *P*_ _= 0.858) while when domatia were open, GPM inoculations resulted in increased egg densities (t_28 _= −3.42; *P*_ _= 0.0001). This increase in egg abundance was not only due to increased abundance of females but also oviposition rate as suggested by enhanced number of eggs per female [“GPM−Dom intact”: 1.28 ± 0.14 (mean ± standard error) eggs/female; “No GPM - Dom intact”: 0.82 ± 0.07 eggs/female; “GPM - Dom blocked”: 0.40 ± 0.03 eggs/female; “No GPM - Dom blocked”: 0.38 ± 0.03 eggs/female]. The number of eggs per females was higher in open domatia treatments (GPM: χ^2^_ _= 0.31, *P*_ _= 0.584; Domatia: χ^2^_ _= 7.39, *P*_ _= 0.014; Domatia*GPM: χ^2^_ _= 0.51, *P*_ _= 0.520). The persistence rate of *A. andersoni* was independent of experimental factors (Table 3; Fig. 6).

## Discussion

The results obtained here show that the populations of both predatory mites responded positively to GPM inoculations with an increase in their density. Previous investigations have indicated a positive association between predatory mites populations increases and the occurrence of *Plasmopara viticola* (Berk. et Curtis ex. de Bary) Berlese and De Toni foliar symptoms in vineyards^32^. Laboratory studies showed that the mycelium of this fungus is an alternative food source for predatory mites^39^. In a previous laboratory study, GPM was shown to be an adequate food source for the survival and the development of *T. pyri* and *A. andersoni*^34^. Our experiment indicates that the addition of GPM promoted predatory mite persistence when prey was virtually absent. This aspect was particularly important for *T. pyri.* Availability of GPM enhances the chances for its persistence on vines. *Amblyseius andersoni* persisted independently of GPM but showed higher population levels in presence of this food source.

The presence of domatia was the main factor in promoting the abundance of predatory mites, and their egg laying. For *T. pyri,* the reduced population abundance induced by blocked domatia resulted in a reduction in its overall persistence on plants. Domatia may benefit the mites through several mechanisms including protection against insect predators and possibly desiccation, reduce cannibalism, provide sites for egg-laying (nurseries) and moulting and the capture of alternative food sources transported by wind such as pollen and fungal spores^20^,^21^,^22^,^23^,^24^,^40^,^41^,^42^. Assuming leaf GPM symptoms are positively correlated with deposition of fungal spores on leaves, one can suggest that domatia can retain a higher amount of GPM spores. This relationship provides an additional means by which domatia may increase the concentration of food sources for generalist predatory mites on leaves. Previous investigations evidenced that the pollen amount present on leaves is correlated with the number of domatia on leaf surface^43^.

We found a positive effect of domatia on the abundance of predatory mite eggs. Results on eggs/female ratio suggest an effect of domatia on *A. andersoni* oviposition, but this was not the case for *T. pyri*. Domatia represented a key factor positively influencing the abundance of *A. andersoni* on grape leaves. *Typhlodromus pyri* was also favored by domatia and showed positive response to hairiness along leaf veins in contrast with *A. andersoni*^21^,^44^,^45^. This may explain some of the differences between the two predatory mites observed in this study, since the leaves of Baco Noir grapevine have relatively large domatia and few trichomes along the veins^25^. Domatia represented the principal oviposition sites for *A. andersoni* and when these were blocked its oviposition was reduced. On the other hand, when domatia were blocked, *T. pyri* used leaf hairs along veins as preferred oviposition sites (Pozzebon, pers. observation).

The interactive effect of domatia and GPM studied here showed different outcomes between the two predatory mites. We found an additional interaction effect of GPM and domatia only for *A. andersoni* egg abundance (Fig. 3). In terms of eggs/female ratio, however, *A. andersoni* responded positively to the availability of domatia but not to GPM. In treatments with open domatia and GPM the number of *A. andersoni* females and the egg/female ratio were slightly higher as compared to other treatments. Therefore the interactive effect of GPM and domatia for egg abundance was driven by a combination of both the increased abundance of females and increased oviposition rate per female. A different response to the interactive effect of GPM and domatia was found for *T. pyri*: the availability of GPM attenuated unfavorable leaf traits (i.e. lack of domatia) promoting its abundance and persistence on plants. As mentioned before domatia have a function as traps for wind-borne food resources for predatory mites. In blocked domatia treatment, predatory mites are likely to encounter food limitation. In our experiment we added a supplementary food resource represented by GPM mycelium. Based on the results obtained here, we suggest that the concentration of this resource was enough to allow the retention of *T. pyri* on leaves with blocked domatia. The same effect was not observed for *A. andersoni*. Indeed the latter have higher nutritional requirements than *T. pyri*, and *A. andersoni* showed a lower survival rate when fed GPM than *T. pyri*^34^. Thus the lower concentration of GPM mycelium, and in general of food resources, in blocked domatia treatments was probably not adequate to sustain high population numbers of *A. andersoni*.

Even more than the effects of access to domatia and GPM inoculations, the presence of *A. andersoni* resulted in the strongest effect on *T. pyri* abundance on plants. In mixed species releases, *T. pyri* abundance and persistence rates were consistently low whereas *A. andersoni* was unaffected by the presence of the competitor indicating an asymmetric relationship in these interspecific interactions. Releases of *A. andersoni* reduced *T. pyri* populations independently of domatia availability. Somewhat counter to these findings, in another study where predatory mites were considered as intraguild prey, domatia protected them from higher order predation^21^ and in general, habitat structural complexity attenuates IGP favoring predator coexistence^12^,^13^,^46^. Intraguild predation is often a size-dependent interaction, with the IG-predator being larger in size than the IG-prey^1^,^47^,^48^. Different size among interacting species is a key aspect that influences the effect of leaf structural surface complexity on IGP involving predatory mites: leaf pubescence can protect small IG-prey from large IG-predator^46^,^49^. In our experiment both IG-predator and IG-prey inhabit the same microhabitats and have a high probability of encounter that hampers the species with the lower capability for interspecific predation. A similar phenomenon has been observed on cannibalism among *Pardosa* spiders, where in structurally complex habitat cannibalistic interactions are diminished only between stages of different body-size classes (large on small), but not on those of the same body–size class^12^. However, previous studies performed on coffee plants (with pit-shaped domatia) and sweet pepper plants (hair tuft domatia), found that domatia reduced IGP among predatory mites^50^. Here we provide evidence in the different direction, since no effect of domatia was found on IGP in our case. This difference may be explained by the type of domatia involved and relative size differences between interacting species. In our case, domatia of grapevine are constituted by dense tufts of non-glandular trichomes (hairs) that are different from the pit-shaped domatia of coffee plants^18^. The differences in body size between *T. pyri* and *A. andersoni* are lower than those between the species involved in the previous study. We hypothesize that the domatia of grapevine provide less restriction to access of both predatory mites and thus not result in a reduction of encounters. However the mechanism that regulates the effect of domatia on IGP among predatory mites needs further study that investigates the interactions among mites within domatia, as suggested by Ferreira *et al.*^50^.

In our experiment the food web was comprised of two predatory mites species engaged in IGP and a supplemental food resource represented by GPM. The supplemental food resource had an effect on population increase of individual species but no effect was observed on the interaction between the two predatory mites. However, it should be noted that in the treatment with IGP the availability of GPM resulted in a relatively higher persistence rate of *T. pyri*, while in absence of the fungus no immatures were found on vines. Theoretical predictions on the positive effect of trophic supplements on predator coexistence are based on the assumption that the predators can maintain positive growth just on the supplemental food source^51^. In a previous laboratory experiment neither of the two predatory mite species used in this study were able to reproduce on GPM and thus we considered this food as a low quality resource^34^. Our experiment suggests that diet enrichment with a low quality food source had a limited effect on IGP. Theoretical models predict that only high quality alternative food sources with a differential effect on predators can influence IGP, resulting in increased coexistence of predators. Based on our results we can also suggest that a low quality supplemental food source can favor coexistence but at a low IG-prey population level.

Results of this research have implications for understanding the community population dynamics of generalist predators in perennial plant systems where persistence is the key to successful biological control and factors that enhance mite habitat suitability are important. For phytoseiids, the generalist feeding habit is a fundamental requirement for persistence and thus for successful biological control of herbivore mites^52^. Alternative food sources are often temporally limited, thus the ability to forage on a wide range of foods, including GPM or other fungi, enhances the persistence in the system. Feeding on multiple species increases community stability^53^,^54^ and generalist feeding behavior can determine long-term survival that is favorable in evolutionary processes that occur in systems characterized by trade-offs of different food sources^15^. Our results suggest that supplemental food can increase the abundance and persistence of predators. An additional contribution to the success of generalist phytoseiid mites is provided by domatia and more generally, by habitat structural complexity that enhances top-down impact on plant exploiters. However, in some cultivars of *Vitis vinifera* L. domatia are small or non-existent^36^,^43^,^55^ and thus availability of alternative food source such as mildews are likely to attenuate unfavorable condition for natural enemies.

Our findings suggest that structural and nutritional diversity/complexity can help maintain associations between beneficial mites and plants.

This study confirmed that IGP can have consistent impacts on predatory mite community composition^37^,^48^,^56^. Despite the absence of significant effects of domatia and GPM on IGP, it is noteworthy that under IGP, in the treatment without GPM and with blocked domatia, the persistence of *T. pyri* was low and no immatures were found on leaves. These results strongly suggest that in this situation *T. pyri* will have a high probability of going locally extinct over time. Thus despite the marginal influence of supplemental food source and habitat structural complexity on IGP found here, we can suggest that these factors favored the coexistence of predators at low population levels of IG-prey. Moreover the results of this study highlight that the impact of supplemental food sources and habitat structural complexity on coexistence of predators engaging in IGP depends on the quality of supplemental food and the degree of difference in body size of interacting species.

## Methods

### Stock cultures

*T. pyri* and *A. andersoni* individuals used in experiments were obtained from laboratory colonies reared at 25 °C and 70 ± 10% of relative humidity. Phytoseiids were reared on grape leaves of Baco Noir variety, a *Vitis vinifera x Vitis riparia* hybrid or on bean leaves (*Phaseolus vulgaris* L.) infested with two-spotted spider mite *Tetranychus urticae* Koch. Two-spotted spider mites were reared on bean grown under glasshouse conditions. A GPM colony, used as a source of inoculum for field experiments, was maintained on potted “Baco Noir” grapevines growing in a walk-in growth chamber (20–30 °C and 70–80% RH).

### Experimental design

The impact of GPM and domatia on phytoseiid abundance and interspecific interactions was evaluated in an experiment conducted on potted grapevines placed outdoors at Cornell University’s New York State Agricultural Experiment Station (NYSAES) in Geneva, NY USA. To evaluate the effects of GPM and domatia on IGP we released the two species of phytoseiids on selected leaves singly or in combination (i.e. *T. pyri*, *A. andersoni* and *T. pyri* + *A. andersoni*) according to an additive design, doubling the total density of mites where both species were released. An additive design is appropriate to study interspecific interactions since predator diversity effects are not confounded with changes in intraspecific interactions^5^,^6^,^13^,^57^. Moreover an increase in abundance of predatory mites on grapevines is often correlated with an increase in their diversity^31^,^32^. Hence, we considered that changes in abundance of each predatory mite species was affected by variation in the strength of interspecific interaction (i.e., intraguild predation). We used Baco Noir vines which are characterized by having well-developed leaf domatia^29^.

The two-factorial experimental design comprised 4 treatments for each phytoseiid combination (Table 4). Each combination was applied to 8 vines. The experiment was a completely randomized block design with 4 blocks of 24 plants each. On each block, treatment combinations were replicated twice. Cane cuttings of Baco Noir were obtained from an experimental vineyard on the NYSAES campus during the previous winter. Dormant cuttings were initially rooted in moist Perlite^®^ in the greenhouse and subsequently transplanted to individual pots (4 l) containing a mixture of sand, peat moss, and Vermiculite^®^. Vines were moved from the greenhouse to an experimental field when three leaves were fully developed. The field was regularly mowed to minimize pollen flow. Vines were watered as needed and fertilized approximately weekly with a complete fertilizer solution (Peter’s Professional 20-20-20 plus micronutrients [Scotts, Marysville, Ohio, USA]). Osmocote^®^ (Scotts, Marysville, Ohio; ≈1,5 g per pot) was applied once when pots were moved outdoors. Vines were pruned to a single growing shoot and trained to a bamboo stake to prevent adjacent vines from touching. When plants had at least 5 mature leaves GPM was inoculated by spraying a distilled water suspension of conidia obtained from laboratory colonies following procedures described in^58^. A second inoculation was made ten days later. Six days after the second inoculation, domatia were blocked by plugging all major vein axils of 5 leaves of each vine with pruning tar (Tree-Cote by Walter E. Clark and Son, Orange, Connecticut). For vines assigned to the open domatia treatment, pruning tar was placed adjacent to each major vein axil. Pruning tar has been previously used to block access to tuft-form domatia without injuring the plant^55^,^58^,^59^. Predatory mites were released one week later. Each vine received 8 adult females of the appropriate species. Predatory mites were first moved from the laboratory colony to a 1 cm diameter leaf disk (four predators per disk), then transported to the field and attached to the second and forth leaves using a metal pin. Prior to the mite release, Stickum^®^ (Tangle-foot Company, Grand Rapids, MI, USA) was applied to vine shoots and stake above the fifth assay leaf and below the first assay leaf to restrict movement of mites to the five experimental leaves. The experiment lasted for one month, enough time for the development of about three generations at similar environmental conditions^34^. At the end of the experiment, assay leaves were collected from each vine and returned to the laboratory where the number of motile forms and eggs of predatory mites were determined using a dissecting microscope. Some specimens of phytoseiids were mounted on slides, in Hoyer’s medium, and identified under a phase contrast microscope to confirm their identity. Specific keys in literature and slides containing juveniles of vineyard-collected phytoseiids, previously identified by taxonomists, were used for the identification of phytoseiid species. At the same time we estimated the percentage of the bottom side of each leaf covered with GPM mycelium using a 1 cm^2^ scale printed on a transparency.

### Pesticide applications

Vines not assigned to be inoculated with GPM were protected from natural GPM infections or from secondary exposure to inoculated vines by treating them with fungicides, once with azoxystrobin (100 ml/hl) and twice with myclobutanil (60 ml/hl). An additional fungicide application was made with metalaxyl to protect grapevines from Grape Downy Mildew (*Plasmopara viticola*) infections. The miticide hexythiazox (50 g/hl) was also applied to all vines before phytoseiid releases in order to exclude the presence of Tydeid mites because of their effects on GPM^55^. Pesticides used were not detrimental to phytoseiids (Loeb unpublished data).

### Statistical analysis

We used a three-way mixed model ANOVA to analyze the effect of GPM inoculation, domatia, IGP and their interactions, on phytoseiid populations. In modeling we considered GPM inoculation (Y/N), domatia access (Y/N), presence of interspecific competitors (Y/N) and all their interactions as fixed effects factors. Block was considered a random effects factor. Analyses were carried out using PROC MIXED of SAS (v 9.2, SAS Institute, Cary, NC), with REML (Restricted Maximum Likelihood) parameter estimation and applying a t-test to examine differences in least-squares means (α_ _= 0.05). Random factors were evaluated by a Likelihood ratio test on the difference between models that differ in their random effects structure under the null hypothesis of zero variance. This test statistic follows a χ^2^-distribution and in this case with a single degree of freedom. In a first set of analyses we used the model described above to evaluate the effect of experimental factors and their interactions on population density (mites per leaf) of predatory mite. Fixed effects were evaluated with an F test (α = 0.05). A second set of analyses using the above described model was performed to evaluate the effect of experimental factors on the persistence of the two predatory mite species during the experiment. As response variable, for each treatment and predatory mite species we considered the ratio (persistence ratio) between the number of vines (replication) with presence of *T. pyri* or *A. andersoni* over the total number of vines where these species were released. The effect of experimental factors and their interactions were evaluated with a χ^2^test (α_ _= 0.05). Because it was not possible to distinguish the egg species, only data from single species treatments were analyzed. A two-way mixed model ANOVA was used with GPM inoculation, domatia manipulation and their interaction as fixed effect factors (F test, α_ _= 0.05) while block was considered a random effects term (χ^2^ test, α_ _= 0.05). Furthermore, to evaluate differences in GPM establishment we analyzed the surface of the under side of the leaf covered with GPM mycelium using two-way mixed model ANOVA, with GPM inoculation and domatia manipulation as fixed effect factors (F test, α_ _= 0.05) and block as a random effects factor (χ^2^test, α_ _= 0.05). Prior to the analysis all data were examined for normality and homoscedasticity and to meet the assumptions for ANOVA. Data on the persistence rate were angular transformed (arc-sine, square root), data on population density, eggs abundance and GPM mycelium were log (x + 1) transformed although graphics are based on untransformed data. Degrees of freedom for all F and t tests were estimated using the Satterthwaite approximation. Moreover all t tests were adjusted for multiple comparisons using Bonferroni method. Since the strength and direction of IGP can be dependent on age and size^1^,^48^,^60^ we evaluated the effect of experimental factors on the different life stages of mites as well as on overall predatory mite populations, but graphics showed data on females and immatures. Mean oviposition rates (number of eggs per female) were compared using a generalized linear mixed model (GLMM) with a binomial distribution logit function and a χ^2^ test (α_ _= 0.05) was used to evaluate the effect of GPM inoculation, domatia access.

## Additional Information

**How to cite this article**: Pozzebon, A. *et al.* Role of supplemental foods and habitat structural complexity in persistence and coexistence of generalist predatory mites. *Sci. Rep.* **5**, 14997; doi: 10.1038/srep14997 (2015).

## Figures and Tables

**Table 1:** Results of mixed model analysis of variance with GPM foliar symptoms measured at the end of the experiment as the dependent variable and domatia condition and GPM inoculation as fixed effect independent variables.

Grape powdery mildew symptoms				
Source of variation	d.f.	F	χ2	p
Fixed				
Grape powdery mildew (GPM) inoculations	1, 87	1112.19		<0.001
Domatia	1, 87	13.15		<0.001
GPM*Domatia	1, 87	13.22		<0.001
Random				
Block		1	1.51	0.219

Block was treated as a random effect in the model. Degrees of freedom in all models were calculated using the Satterthwaite approximation.

**Table 2:** Mixed model analysis of variance statistics for the experiment examining the influence of access to domatia, presence of GPM and IGP (fixed) effects on phytoseiids abundance observed at the end of the experiment.

Source of variation	*Typhlodromus pyri*				*Amblyseius andersoni*			
	d.f.	F	χ2	p	d.f.	F	χ2	p
Motile forms								
Fixed								
Intraguild predation (IGP)	1, 55	71.18		<0.001	1, 51.3	2.09		0.155
Grape powdery mildew (GPM)	1, 55	5.59		0.021	1, 51.3	4.40		0.041
Domatia	1, 55	31.45		<0.001	1, 51.3	27.10		<0.001
IGP*GPM	1, 55	1.73		0.193	1, 51.3	1.13		0.293
IGP*Domatia	1, 55	5.45		0.023	1, 51.3	0.08		0.780
GPM*Domatia	1, 55	5.92		0.018	1, 51.3	0.04		0.834
IGP*GPM*Domatia	1, 55	1.51		0.224	1, 51.3	0.76		0.387
Random								
Block	1		1.52	0.176	1		0.72	0.396
Females								
Fixed								
Intraguild predation (IGP)	1, 55	45.40		<0.001	1, 55	0.18		0.672
Grape powdery mildew (GPM)	1, 55	6.56		0.013	1, 55	3.04		0.086
Domatia	1, 55	18.21		<0.001	1, 55	10.83		0.002
IGP*GPM	1, 55	3.38		0.071	1, 55	0.01		0.954
IGP*Domatia	1, 55	2.53		0.117	1, 55	1.23		0.271
GPM*Domatia	1, 55	0.40		0.531	1, 55	0.86		0.358
IGP*GPM*Domatia	1, 55	0.09		0.768	1, 55	0.01		0.936
Random								
Block	1		3.75	0.052	1		2.14	0.144
Immatures								
Fixed								
Intraguild predation (IGP)	1, 52.4	46.87		<0.001	1, 56	3.32		0.073
Grape powdery mildew (GPM)	1, 52.4	4.60		0.036	1, 56	2.90		0.094
Domatia	1, 52.4	17.95		<0.001	1, 56	16.39		<0.001
IGP*GPM	1, 52.4	1.76		0.190	1, 56	1.72		0.195
IGP*Domatia	1, 52.4	4.80		0.030	1, 56	0.01		0.975
GPM*Domatia	1, 52.4	6.85		0.011	1, 56	0.15		0.704
IGP*GPM*Domatia	1, 52.4	2.24		0.140	1, 56	0.50		0.482
Random								
Block	1		0.51	0.475	1		0.02	0.887
Eggs								
Fixed								
Grape powdery mildew (GPM)	1, 28	1.73		0.198	1, 28	6.48		0.017
Domatia	1, 28	7.94		0.008	1, 28	38.43		<0.001
GPM*Domatia	1, 28	0.81		0.377	1, 28	5.25		0.029
Random								
Block	1		0.05	0.823	1	0.01		0.933

We used restricted maximum likelihood methods. Block was considered a random effect. Degrees of freedom in all models were calculated using the Satterthwaite approximation.

**Table 3:** Mixed model analysis of variance statistics for experiment examining the influence of access to domatia, presence of GPM and IGP (fixed) effects on phytoseiids persistence rate observed at the end of the experiment.

Source of variation	*Typhlodromus pyri*		*Amblyseius andersoni*	
	χ2	p	χ2	p
Motile forms				
Fixed				
Intraguild predation (IGP)	15.98	<0.001	0.33	0.564
Grape powdery mildew (GPM)	4.00	0.046	0.33	0.564
Domatia	7.10	0.008	0.33	0.564
IGP*GPM	0.44	0.505	0.33	0.564
IGP*Domatia	0.00	1.000	0.33	0.564
GPM*Domatia	4.00	0.046	3.00	0.083
IGP*GPM*Domatia	0.44	0.505	0.33	0.564
Random				
Block	3.50	0.060	0.20	0.654
Females				
Fixed				
Intraguild predation (IGP)	15.79	<0.001	0.50	0.480
Grape powdery mildew (GPM)	0.84	0.359	0.50	0.480
Domatia	11.30	0.001	0.50	0.480
IGP*GPM	0.09	0.763	2.00	0.157
IGP*Domatia	0.09	0.760	2.00	0.157
GPM*Domatia	0.84	0.359	2.00	0.157
IGP*GPM*Domatia	0.09	0.760	0.50	0.480
Block	0.90	0.343	0.10	0.752
Immatures				
Fixed				
Intraguild predation (IGP)	8.70	0.003	2.00	0.157
Grape powdery mildew (GPM)	5.83	0.016	0.50	0.480
Domatia	8.70	0.003	2.00	0.157
IGP*GPM	0.07	0.789	0.50	0.480
IGP*Domatia	1.80	0.180	2.00	0.157
GPM*Domatia	5.83	0.016	0.50	0.480
IGP*GPM*Domatia	0.07	0.789	0.50	0.480
Random				
Block	0.10	0.752	0.10	0.752

We used restricted maximum likelihood methods. Block was considered a random effect. Degrees of freedom in all models were equal to 1.

**Table 4:** Description of treatments used in the experiment to examine the influence of GPM, access to domatia, and presence or absence of a competitor.

Phytoseiid species released	Treatments	GPM	*Domatia*
*Typhlodromus pyri*	GPM *Domatia* intact	+	+
	GPM *Domatia* blocked	+	−
	No GPM *Domatia* intact	−	+
	No GPM *Domatia* blocked	−	−
*Amblyseius andersoni*	GPM *Domatia* intact	+	+
	GPM *Domatia* blocked	+	−
	No GPM *Domatia* intact	−	+
	No GPM *Domatia* blocked	−	−
*Typhlodromus pyri + Amblyseius andersoni*	GPM *Domatia* intact	+	+
	GPM *Domatia* blocked	+	−
	No GPM *Domatia* intact	−	+
	No GPM *Domatia* blocked	−	−

The symbol (+) means that GPM was inoculated or that domatia were left open. The symbol (−) means that GPM was not inoculated or domatia were blocked.
